# Supplementary material for: Providing environmental enrichments can reduce subclinical spondylolisthesis prevalence without affecting performance in broiler chickens
Source: PLoS One. 2023 Apr 7;18(4):e0284087. doi: 10.1371/journal.pone.0284087 (PMC10081764; doi:10.1371/journal.pone.0284087)
Supplement: Attachment — Submitted filename: Response to Reviewers.docx [file pone.0284087.s001.docx]

Botucatu, February 22, 2023

Editors-in-Chief PlosOne

Dear Editor,

We are very happy to receive such clear and important suggestions for improving our manuscript. We applied the corrections suggested by the reviewer, reviewed typos, clarified points that seemed unclear, and adjusted some other details as requested. The figure files were uploaded to the Preflight Analysis and Conversion Engine (PACE) digital diagnostic tool, following the journal requirements. Our detailed responses are shown in blue below.

We look forward to receiving your decision.

**Journal requirements:**

1. Please ensure that your manuscript meets PLOS ONE's style requirements, including those for file naming. The PLOS ONE style templates can be found at

<https://journals.plos.org/plosone/s/file?id=wjVg/PLOSOne_formatting_sample_main_body.pdf> and 
<https://journals.plos.org/plosone/s/file?id=ba62/PLOSOne_formatting_sample_title_authors_affiliations.pdf>

Thank you for your suggestion. We double-checked our manuscript to ensure it meets Plos One’s style requirements.

2. To comply with PLOS ONE submissions requirements, in your Methods section, please provide additional information regarding the experiments involving animals and ensure you have included details on (1) methods of sacrifice, (2) methods of anesthesia and/or analgesia, and (3) efforts to alleviate suffering.

Thank you for your suggestions. We double-checked and ensured that this information was added to the manuscript.

(1) Methods of sacrifice: “Chickens were fasted for 8 hours. Fasting is a common procedure prior to processing to allow emptying of the gastro-intestinal tract and is required by the Brazilian Ministry of Agriculture for food safety considerations (SDA/MAPA Ordinance No. 365, of 16 July 2021). The chickens were weighed and then stunned using an electric stunner (Fluxo UFX 7, Chapecó, SC, Brazil). The chickens were exsanguinated via a cut to the carotid artery and jugular vein.” (L199-204)

(2) Methods of anesthesia and/or analgesia: Not applicable to this study.

(3) Efforts to alleviate suffering: “Chickens presenting GS 2 were euthanized as a humane endpoint, and their scores were noted.” (L193-194).

3. We note that the grant information you provided in the ‘Funding Information’ and ‘Financial Disclosure’ sections do not match.

When you resubmit, please ensure that you provide the correct grant numbers for the awards you received for your study in the ‘Funding Information’ section.

Thank you for letting us know. We ensured that the grant information matched both sections.

4. We note that you have indicated that data from this study are available upon request. PLOS only allows data to be available upon request if there are legal or ethical restrictions on sharing data publicly. For more information on unacceptable data access restrictions, please see <http://journals.plos.org/plosone/s/data-availability#loc-unacceptable-data-access-restrictions>.

In your revised cover letter, please address the following prompts:

a) If there are ethical or legal restrictions on sharing a de-identified data set, please explain them in detail (e.g., data contain potentially sensitive information, data are owned by a third-party organization, etc.) and who has imposed them (e.g., an ethics committee). Please also provide contact information for a data access committee, ethics committee, or other institutional body to which data requests may be sent.

b) If there are no restrictions, please upload the minimal anonymized data set necessary to replicate your study findings as either Supporting Information files or to a stable, public repository and provide us with the relevant URLs, DOIs, or accession numbers. For a list of acceptable repositories, please see <http://journals.plos.org/plosone/s/data-availability#loc-recommended-repositories>.

We will update your Data Availability statement on your behalf to reflect the information you provide.

Thank you for your suggestion. We will update our Data Availability statement. “The datasets generated and analyzed during the current study are available in http://dx.doi.org/10.17632/xp62w777ks.1”

5. Please include captions for your Supporting Information files at the end of your manuscript, and update any in-text citations to match accordingly. Please see our Supporting Information guidelines for more information: <http://journals.plos.org/plosone/s/supporting-information>.

Thank you for your suggestion. We added captions of support information at the end of the manuscript.

6. Please review your reference list to ensure that it is complete and correct. If you have cited papers that have been retracted, please include the rationale for doing so in the manuscript text, or remove these references and replace them with relevant current references. Any changes to the reference list should be mentioned in the rebuttal letter that accompanies your revised manuscript. If you need to cite a retracted article, indicate the article’s retracted status in the References list and also include a citation and full reference for the retraction notice.

Thank you for your suggestion. We double-checked our reference list to ensure that everything is correct.

**Reviewer #1**: Comments:

Generally I find this experiment to be a nice and well conducted study and well presented in the manuscript. I have only minor comments and suggestions as follows:

**Reviewer #1**: Line 75: "When providing diets diluted in fiber in ...". Please specify type of fiber.

Thank you for your suggestion we added that information: “When providing diets diluted with wood sawdust fiber in the first week of life, prevalence of subclinical spondylolisthesis was reduced but daily gain was also stunted [16].” (L76-78)

**Reviewer #1**: Line 83: Change "favor" to improve or enhance.

Thank you for your suggestion, we modified as suggested: “Environmental enrichments increase the complexity of broiler chicken environments and enhance the expression of natural behaviors” (L83-84).

**Reviewer #1**: Line 90: Delete "beneficial" as it means the same as "enrichments".

Thank you for your comment. We understand the point of view of the reviewer. However, environmental enrichments can be ineffective if animals do not use them or if natural behaviors are not stimulated/abnormal behaviors not reduced. Although the objective of enrichments is always for some kind of benefit, this is not always the case. Therefore, we decided to keep the term ‘beneficial’ in the sentence. A valuable review on this topic can be found by Riber et al. 2018 (http://dx.doi.org/10.3382/ps/pex344), which details a range of enrichments used for broiler chickens and their effects (positive, negative, or no effect) on leg health, behavior, and performance parameters. [no edit]

**Reviewer #1**: Line 173: Change "During recording days, only daily health checks were performed to ..." to "Health checks were only performed during recording days in order to ...".

Thank you for your suggestion, edited as recommended: “Human disturbance was limited on recording days, and birds were only disturbed for health checks. (L174-175)”

**Reviewer #1**: Line 190: Change "difficulty" to difficulty in".

Thank you for your suggestion, edited as suggested: “…score 1 (GS 1) was attributed to chickens that exhibited difficulty in walking in a way that was easily identifiable through observation…” (L190-192).

**Reviewer #1**: Line 196: Please explain why the chickens were fasted for 8 hours before killing.

The authors followed pre-slaughter management standards for broilers routinely adopted in commercial farms. The Brazilian Ministry of Agriculture (MAPA) regulations establish 12 hours as the maximum pre-slaughter fasting period for broilers (recommended between 6 to 12 hours) (Pereira et al. 2013, http://dx.doi.org/10.1590/S1516-635X2013000200007). This motivation was clarified in the manuscript: “Fasting is a common procedure prior to processing to allow emptying of the gastro-intestinal tract and is required by the Brazilian Ministry of Agriculture for food safety considerations (SDA/MAPA Ordinance No. 365, of 16 July 2021).” (L199-202).

**Reviewer #1**: You write that "The chickens were weighed and then stunned using an electric stunner (Fluxo UFX 7, Chapecó, SC, Brazil). Are you sure that the electric stunning and muscle contractions is not the reason the deforming the cervical spines and nerves? Please add a comment on this.

Thank you for your comment. It is unlikely that the electrical stunning causes subclinical spondylolisthesis. The prevalence of spondylolisthesis may be explained by the anatomical structure of the chicken vertebral column. It is acknowledged that most thoracic and lumbosacral vertebrae fuse to form two immobile units, the *notarium* and the *synsacrum*. This process is normally completed at the beginning of sexual maturity (please see: Baumel and Witmer, 1993; Dinev, 2014). That is why there is the assumption that the remaining mobile Т4 and the adjacent vertebrae (including T6) are most prone to development of abnormalities (disturbed bone growth) during the period of skeletal immaturity. It means this is a long development process. Previous literature reporting on the effects of electrical stunning have not reported vertebrae dislocation as a consequence (Gregory, 1989). Thus, we are confident it cannot be caused instantly by electrical stunning. [no edit]

Baumel, J. and Witmer, L. (1993) Osteologia, in: Baumel, J., King, A., Breazile, J., Evans, H. & Vanderberge, J. (Eds) Handbook of avian anatomy: nomina anatomica avium, second ed., pp. 56-57 (Nuttall ornitological club, Harvard University, Cambridge, Massachusetts).

Dinev, I. (2014). Axial skeleton pathology in broiler chickens. World's Poultry Science Journal, 70(2), 303-308.

Wilkins, L. J., Gregory, N. G., Wotton, S. B., & Parkman, I. D. (1998). Effectiveness of electrical stunning applied using a variety of waveform-frequency combinations and consequences for carcase quality in broiler chickens. British poultry science, 39(4), 511-518.

**Reviewer #1**: Line 205 and a general comment to the study: Were the same chickens observed clinically and compared to the pathology findings after killing? This could be very valuable information and also add to the strength of the posed causal relationship between pathology of the cervical spines and affected gait. Please comment on this.

Thank you for your comment. Yes, they were. Prior to slaughter, chickens’ gait scores were assessed. However, the subclinical spondylolisthesis prevalence in this study was not associated with gait scores, which is in line with expectations of a subclinical rather than a clinical diagnosis. A correlation analysis between gait and subclinical spondylolisthesis was added to the manuscript:

Methods: “Before gait assessment at 42 days of age, 48 chickens of each treatment (12 per pen) were arbitrarily selected and leg banded for subclinical spondylolisthesis prevalence assessment. Then, gait assessment proceeded in all chickens.” (L194-196)

“The correlation between gait score and subclinical spondylolisthesis prevalence was assessed using Spearman’s correlation analysis with the CORR procedure, considering a significance of P < 0.05.” (L238-240)

Results: “Gait was not associated with subclinical spondylolisthesis prevalence (r = 0.010, P = 0.888).” (L292-293).

**Reviewer #1**: Table 2: Why is performance not stratified according to week of age? This will add better information than the not mutually exclusive grouping of 1-21, 1-35 and 1-42.

Thank you for your comment. We understand the comment but would argue to keep the results as they are. Performance later in life cannot be independent from performance earlier in life (meaning performance between day 21-35 is impacted by performance between day 1-21). We kindly invite the reviewer to look at the following published papers in high impact factor journals (including Plos One) that used the same way to present performance data:

Ptak A, Bedford MR, Świątkiewicz S, Żyła K, Józefiak D (2015) Phytase Modulates Ileal Microbiota and Enhances Growth Performance of the Broiler Chickens. PLoS ONE 10(3): e0119770. https://doi.org/10.1371/journal.pone.0119770

Liu Z-Y, Wang X-L, Ou S-Q, Hou D-X, He J-H (2020) Sanguinarine modulate gut microbiome and intestinal morphology to enhance growth performance in broilers. PLoS ONE 15(6): e0234920. https://doi.org/10.1371/journal.pone.0234920

Sizmaz, O., Barrett, N. W., Lewis, J., Yakout, H., & Persia, M. E. (2022). Effect of various concentration of butyric acid on growth performance, intestinal lesion scores, and body composition of broilers raised on used litter. Journal of Applied Poultry Research, 31(4), 100296.

Jacobs, L., Persia, M. E., Siman-Tov, N., McCoy, J., Ahmad, M., Lyman, J., & Good, L. (2020). Impact of water sanitation on broiler chicken production and welfare parameters. Journal of Applied Poultry Research, 29(1), 258-268.

Paz, I. C. D. L. A., de Lima Almeida, I. C., de La Vega, L. T., Milbradt, E. L., Borges, M. R., Chaves, G. H. C., ... & Andreatti Filho, R. L. (2019). Productivity and well-being of broiler chickens supplemented with probiotic. Journal of Applied Poultry Research, 28(4), 930-942.

**Reviewer #1**: Other suggestions:

As the authors use many abbreviations, I suggest a list of abbreviations is added to the manuscript. When reading a publication, it is always very tiring to spend time on finding (re-finding) the definitions of the abbreviations after the first presentation.

Thank you for your suggestion. We omitted as many abbreviations as possible to make the reading more fluid.

**Reviewer #1**: Some of the environmental enrichments have a statistically significant effect; however, the factual improvement seems to be minor. The manuscript will benefit if the authors add a comment or some estimations of the attributable effects of these environmental enrichments; I think they will be minor, and this will be a nice contribution the manuscript to underline, that this type of "problem" (spondylolisthesis) is multifactorial and that many factors each have only a small impact, and which makes it so difficult and complex to solve such problems.

Thank you for your suggestion. The authors agree with the reviewer and a statement underlining the multifactorial causation for subclinical spondylolisthesis prevalence was added to the manuscript: “However, it is important to underline that subclinical spondylolisthesis is a multifactorial locomotor disorder and that many factors, including genetic predisposition, nutrition, environment, age, stocking density, incubation conditions, and bedding material can contribute to the etiology of spondylolisthesis [7,11,13–16]. Our findings suggest that environmental enrichment (step platforms and laser lights) can be used as a tool to reduce subclinical spondylolisthesis prevalence compared to a barren environment (control).” (L440-446).
